# Treatment Allocation and Survival Outcomes in Potentially Resectable Colorectal Liver Metastases: A Real-World Cohort Study from Thailand

**DOI:** 10.3390/cancers18193096

**Published:** 2026-09-23

**Authors:** Warakorn Jaseanchiun, Hiroyuki Kato, Chutima Phettool, Chanchai Suwannakij, Jadsada Athigakunagron, Setthasiri Pantanakul, Papanun Methakittrakoon, Nattapon Passawart, San Wattanasuttivong, Chaninart Liengudom, Karun Sereeborwornthanasa, Panot Yimchareon

**Affiliations:** 1 Department of Surgery, Bhumibol Adulyadej Hospital, Bangkok 10220, Thailand; 2 Department of Gastroenterological Surgery, School of Medicine, Fujita Health University, Bantane Hospital, Nagoya 454-8509, Aichi, Japan; 3 Medical Oncology Unit, Department of Medicine, Bhumibol Adulyadej Hospital, Bangkok 10220, Thailand; 4 Department of Radiology, Bhumibol Adulyadej Hospital, Bangkok 10220, Thailand

**Keywords:** colorectal liver metastases, liver resection, resectability, treatment allocation, hepatobiliary referral, multidisciplinary team, Thailand

## Abstract

Background/Objectives: Liver resection is a potentially curative treatment for colorectal liver metastases (CRLM), but some potentially resectable patients may not reach specialist hepatobiliary assessment. This study evaluated treatment allocation, survival outcomes, and the potential gap between resectability and actual liver resection in a real-world Thai cohort. Methods: We retrospectively reviewed 265 patients with CRLM treated at a tertiary-care hospital in Thailand between 2012 and 2022. Patients were classified into liver resection (LR; *n* = 57) and no liver resection (NLR; *n* = 208) groups. NLR patients without extrahepatic metastases were retrospectively reassessed for potential resectability by two hepatobiliary surgeons using predefined anatomical, oncological, and liver functional criteria. Baseline characteristics and overall survival (OS) were compared between LR patients and potentially resectable NLR patients. Cox regression analysis was also performed in the combined resectable cohort. Results: Median OS was 59 months in the LR group and 23 months in the NLR group. In the NLR group, age < 65 years (adjusted HR 0.663, 95% CI 0.458–0.960; *p* = 0.030) and absence of extrahepatic metastases (adjusted HR 0.637, 95% CI 0.417–0.973; *p* = 0.037) were independently associated with longer OS. Among 67 NLR patients without extrahepatic metastases, 27 (40.3%; 13.0% of all NLR patients) were considered potentially resectable. All 27 had undergone primary colorectal tumor resection but had not been referred for hepatobiliary surgical assessment. No significant differences were observed in the measured baseline characteristics between these 27 patients and the LR group. Median OS was 29 versus 59 months, respectively (log-rank *p* = 0.018). In the combined resectable cohort (*n* = 84), LR remained associated with longer OS after adjustment for timing of liver metastases, number of liver lesions, and bilobar distribution (adjusted HR 0.40, 95% CI 0.20–0.81; *p* = 0.011). Conclusions: A clinically relevant subgroup of non-resected patients met predefined criteria for potential resectability on retrospective specialist reassessment. These findings should be interpreted as real-world treatment-allocation observations rather than evidence of a causal survival benefit of LR and support early hepatobiliary referral and repeated multidisciplinary assessment of resectability.

## Simple Summary

Some patients with colorectal liver metastases may not undergo liver surgery even when resection could be technically possible. We reviewed 265 patients treated at a tertiary-care hospital in Thailand. Among 67 non-resected patients without extrahepatic metastases, 27 (40.3%) were considered potentially resectable after retrospective assessment by two hepatobiliary surgeons. All 27 had undergone resection of the primary colorectal tumor but had not been referred for hepatobiliary surgical assessment. Their measured baseline characteristics did not significantly differ from those of 57 patients who underwent liver resection, but median overall survival was 29 versus 59 months. In the combined cohort of 84 patients considered resectable, liver resection remained associated with longer survival after adjustment for the timing, number, and bilobar distribution of liver metastases. These findings primarily describe real-world treatment allocation and support early hepatobiliary referral and repeated multidisciplinary assessment; the retrospective design does not establish a causal benefit of surgery.

## 1. Introduction

Colorectal cancer frequently metastasizes to the liver, and liver-directed treatment plays a central role in the management of colorectal liver metastases (CRLM). For patients in whom all macroscopic disease can be eradicated while preserving an adequate functional liver remnant, liver resection remains the principal potentially curative treatment. Contemporary guidelines therefore recommend surgery, with or without perioperative systemic therapy, for patients considered candidates for curative-intent treatment and emphasize multidisciplinary assessment involving hepatobiliary surgeons, medical oncologists, radiologists, and other specialists in liver-directed treatment [1,2]. Long-term outcomes after liver resection have improved substantially. A recent nationwide Japanese survey reported a 5-year overall survival (OS) rate of 62.4% after hepatectomy [3], while a large systematic review of international surgical series reported a median 5-year OS of 38%, with outcomes varying considerably among institutions [4].

The concept of resectability has also evolved considerably. The number, size, or bilobar distribution of liver metastases should no longer be regarded as absolute contraindications to surgery. Rather, technical resectability is determined primarily by whether all macroscopic disease can be completely treated while preserving an adequate future liver remnant with sufficient vascular inflow, venous outflow, and biliary drainage [2,5,6]. Consequently, patients with apparently extensive liver disease may still be candidates for curative-intent treatment when assessed by an experienced hepatobiliary surgeon.

Despite these advances, a gap may remain between technical resectability and actual treatment allocation. Previous studies have demonstrated substantial variation in referral practice and have shown that some patients initially managed without curative-intent liver treatment were subsequently considered resectable or potentially resectable after specialist hepatobiliary review [7,8,9,10]. Thus, improving outcomes in CRLM may depend not only on advances in surgical techniques and systemic therapy but also on ensuring that potentially resectable patients are appropriately identified and referred for specialist assessment. This issue may be particularly relevant in healthcare settings where access to dedicated hepatobiliary expertise and formal multidisciplinary liver conferences is not uniform. However, real-world evidence regarding treatment allocation, access to specialist resectability assessment, and potential referral gaps remains limited in Thailand and other Southeast Asian settings.

The present study therefore evaluated a real-world cohort of patients with CRLM treated at a tertiary hospital in Thailand. We first characterized treatment allocation and long-term survival in the entire cohort and evaluated the outcomes of patients undergoing liver resection in the context of internationally reported surgical results. We then retrospectively reassessed patients with liver-limited CRLM who had not undergone liver resection to identify those who fulfilled contemporary criteria for potentially curative hepatectomy. To examine treatment allocation among patients considered resectable, we compared the clinical and tumor characteristics of retrospectively resectable but non-resected patients with those of patients who underwent liver resection and evaluated factors associated with OS in this combined population. By examining not only surgical outcomes but also the potential gap between technical resectability and actual receipt of liver resection, this study aimed to describe opportunities to improve specialist referral and multidisciplinary assessment for patients with CRLM in a real-world Southeast Asian clinical setting.

## 2. Patients and Methods

### 2.1. Study Design and Population

This retrospective cohort study included consecutive adult patients diagnosed with colorectal liver metastases (CRLM) at Bhumibol Adulyadej Hospital between 1 January 2012 and 31 December 2022. The diagnosis of CRLM was based on histopathological confirmation and/or characteristic findings on cross-sectional imaging. Patients were excluded if they had undergone previous liver resection or had received systemic treatment before referral to our institution. The study was approved by the Institutional Review Board of Bhumibol Adulyadej Hospital (IRB No. 53/64), and the requirement for written informed consent was waived because of the retrospective nature of the study.

### 2.2. Treatment Groups and Retrospective Assessment of Resectability

Patients were categorized into the liver resection (LR) group if they underwent curative-intent liver resection during the study period and into the no liver resection (NLR) group if they did not undergo liver resection. Treatment decisions were made according to clinical practice, available specialist expertise, and multidisciplinary processes at Bhumibol Adulyadej Hospital at the time of treatment.

To examine the potential gap between technical resectability and actual treatment allocation, patients in the NLR group without extrahepatic metastases were retrospectively reassessed for potential resectability. Two hepatobiliary surgeons (W.J. and H.K.) jointly reviewed the available cross-sectional imaging findings and the clinical history documented in the electronic medical records, and the final resectability classification was reached by consensus.

Potential resectability was assessed according to the indication criteria for liver resection defined in the Japanese Society for Cancer of the Colon and Rectum (JSCCR) Guidelines 2024 [11]. Patients were considered potentially resectable when all of the following criteria were fulfilled: (1) the patient was considered capable of tolerating surgery; (2) the primary colorectal tumor had been controlled or was considered controllable; (3) all metastatic liver tumors were considered amenable to complete surgical resection; (4) no uncontrolled extrahepatic metastases were present; and (5) adequate liver function was expected to remain after hepatectomy. Because the retrospective reassessment was restricted to NLR patients without extrahepatic metastases, the fourth criterion was fulfilled by definition in the reassessed population. The number, maximum diameter, and bilobar distribution of liver metastases were not used as absolute exclusion criteria for resection. Liver functional reserve and the permissible extent of hepatectomy were assessed according to the Makuuchi criteria, which incorporate the presence or absence of ascites, serum total bilirubin, and the indocyanine green retention rate at 15 min (ICG-R15) [12,13]. When complete removal of all liver metastases would have required a major hepatectomy, or when the anticipated future liver remnant appeared insufficient or borderline on CT, CT volumetry was additionally used to estimate the predicted remnant liver volume [14,15]. Resectability was determined by integrating these functional assessments with the anatomical feasibility of complete tumor removal and the available clinical information.

Patients who fulfilled all five JSCCR criteria on retrospective specialist reassessment were classified as potentially resectable NLR patients. Among the 67 NLR patients without extrahepatic metastases, 27 were classified as potentially resectable. In this study, the term “potentially resectable NLR” refers specifically to NLR patients who fulfilled these criteria on retrospective specialist reassessment, whereas “non-resected” refers to patients who did not undergo liver resection during the study period.

### 2.3. Comparison of Patients Considered Resectable

To further evaluate the potential gap between technical resectability and actual treatment allocation, the 27 retrospectively resectable NLR patients were compared with the 57 patients who underwent liver resection. Baseline clinical and tumor characteristics included age, sex, location of the primary colorectal tumor, synchronous or metachronous presentation of liver metastases, number of liver metastases, maximum tumor diameter, bilobar distribution, extrahepatic disease, and serum carcinoembryonic antigen (CEA) level.

Overall survival was compared between the LR group and the retrospectively resectable NLR group. In addition, the two groups were combined to form a cohort of 84 patients considered resectable, comprising 57 patients who underwent liver resection and 27 patients who were retrospectively considered resectable but did not undergo liver resection. Prognostic factors for OS were evaluated in this combined resectable cohort to examine whether actual receipt of liver resection was associated with survival after accounting for clinical and tumor-related factors.

### 2.4. Outcomes

The primary outcome was overall survival (OS), defined as the interval from the date of histopathological diagnosis to death from any cause or the last follow-up.

Secondary outcomes included the proportion of NLR patients with liver-limited disease who were retrospectively judged to be potentially resectable according to the JSCCR criteria, differences in baseline clinical and tumor characteristics between the LR group and the retrospectively resectable NLR subgroup, factors associated with OS in the combined resectable cohort, factors associated with OS in the overall NLR group, and prognostic factors among patients who underwent liver resection.

### 2.5. Statistical Analysis

Continuous variables were summarized as mean ± standard deviation or median with interquartile range, as appropriate, and categorical variables were reported as numbers and percentages. Baseline characteristics of the LR group and the retrospectively resectable NLR subgroup were compared using Fisher’s exact test for categorical variables and Student’s *t*-test or the Mann–Whitney *U* test for continuous variables, as appropriate. Because of its skewed distribution, serum CEA was log-transformed for Cox regression analysis.

OS was estimated using the Kaplan–Meier method and compared using the log-rank test. Cox proportional hazards regression analysis was used to evaluate factors associated with OS.

For the combined cohort of 84 patients considered resectable, univariate Cox regression analyses were performed for liver resection status and relevant clinical and tumor-related variables. Variables demonstrating a significant association with OS in the univariate analysis were subsequently included in a multivariable Cox proportional hazards model. The multivariable model included liver resection status, synchronous versus metachronous presentation, number of liver metastases, and bilobar versus unilobar disease. There were 44 deaths in this cohort, corresponding to approximately 11 events per variable in the four-covariate model.

A separate multivariable Cox proportional hazards analysis was performed in the NLR group to evaluate factors associated with OS. Cox regression analysis was also performed among patients who underwent liver resection to evaluate postoperative prognostic factors.

Hazard ratios (HRs) with 95% confidence intervals (CIs) were reported. All statistical tests were two-sided, and a *p*-value < 0.05 was considered statistically significant. Median follow-up was estimated using the reverse Kaplan–Meier method, in which deaths were treated as censored observations and patients alive at last follow-up as events. Statistical analyses were performed using IBM SPSS Statistics (versions 29 and 32; IBM Corp., Armonk, NY, USA).

## 3. Results

### 3.1. Patient Characteristics

A total of 265 patients with CRLM were included in the study. Of these, 57 patients (21.5%) underwent liver resection (LR group), whereas 208 patients (78.5%) did not undergo liver resection (NLR group) (Table 1). The proportion of patients with a colon primary was 93.0% in the LR group and 99.0% in the NLR group (*p* = 0.009). Five or more liver metastases were present in 7.0% of the LR group and 73.1% of the NLR group (*p* < 0.001). The largest liver metastasis was ≤5 cm in 77.2% and 56.3% of patients, respectively (*p* = 0.004). Bilobar liver metastases were present in 24.6% of the LR group and 79.3% of the NLR group (*p* < 0.001), and extrahepatic metastases were present in 8.8% and 67.8%, respectively (*p* < 0.001). All 57 LR patients underwent resection of the primary colorectal tumor: 34 (59.6%) underwent simultaneous colorectal and liver resection, while 23 (40.4%) underwent staged resection, with colorectal resection preceding liver resection in all staged cases. Only 5 of the 57 LR patients received neoadjuvant systemic therapy for liver metastases. No patient underwent more than one liver resection during the study period. Other baseline characteristics are shown in Table 1. During follow-up, death occurred in 142 of 265 patients (53.6%) in the entire cohort, including 29 of 57 (50.9%) in the LR group and 113 of 208 (54.3%) in the NLR group. Among the 27 potentially resectable NLR patients, 15 (55.6%) died; accordingly, the combined resectable cohort had 44 deaths. Median follow-up, estimated using the reverse Kaplan–Meier method, was 29 months (95% CI, 25–42 months) for the entire cohort, 58 months (95% CI, 53–87 months) for the LR group, and 22 months (95% CI, 16–25 months) for the NLR group.

### 3.2. Overall Survival

The median OS of the entire cohort was 27 months (95% CI, 21.0–32.9 months) (Figure 1).

Median OS was 59 months (95% CI, 42.74–75.26 months) in the LR group and 23 months (95% CI, 19.21–26.79 months) in the NLR group (log-rank *p* < 0.01) (Figure 2).

The 1-, 3-, and 5-year OS rates were 89.5%, 71.9%, and 49.1%, respectively, in the LR group and 64.4%, 27.4%, and 11.5%, respectively, in the NLR group (all *p* < 0.001) (Table 1).

### 3.3. Retrospectively Identified Potentially Resectable Patients in the NLR Group

Among the 208 patients in the NLR group, 67 had no extrahepatic metastases. Retrospective reassessment identified 27 of these 67 patients (40.3%) as potentially resectable according to the predefined resectability criteria. These patients accounted for 13.0% of the entire NLR group.

Among the 27 potentially resectable NLR patients, the mean age was 67.6 ± 13.4 years, 15 patients (55.6%) were male, and 13 (48.1%) had synchronous liver metastases. Nine patients (33.3%) had a single liver metastasis and 18 (66.7%) had 2–4 metastases; none had ≥5 liver metastases. Twenty-four patients (88.9%) had a maximum tumor diameter ≤ 5 cm, 16 (59.3%) had unilobar disease, and all 27 had no extrahepatic metastases. All 27 had undergone resection of the primary colorectal tumor by the colorectal surgical team, but none had been referred for hepatobiliary surgical assessment before being managed with palliative systemic therapy. Systemic regimens used in this subgroup included capecitabine (Xeloda), XELOX, FOLFOX4, and FOLFIRI. Targeted therapy was not analyzed because its use was uncommon during the study period, and no patient in this subgroup underwent conversion to liver resection. The median serum CEA level was 10.0 ng/mL (IQR, 4.5–30.0 ng/mL).

Baseline characteristics were compared between the 27 potentially resectable NLR patients and the 57 patients in the LR group (Table 2).

Mean age was 67.6 ± 13.4 versus 64.8 ± 11.1 years (*p* = 0.312), male sex was present in 55.6% versus 61.4% (*p* = 0.641), synchronous presentation in 48.1% versus 68.4% (*p* = 0.094), bilobar disease in 40.7% versus 24.6% (*p* = 0.201), and a largest liver metastasis > 5 cm in 11.1% versus 22.8% (*p* = 0.247), respectively. Median CEA levels were 10.0 ng/mL (IQR, 4.5–30.0) and 10.5 ng/mL (IQR, 4.6–49.0), respectively (*p* = 0.958), as shown in Table 2.

Median OS was 29 months (95% CI, 16.02–41.98 months) in the potentially resectable NLR group and 59 months (95% CI, 42.74–75.26 months) in the LR group (log-rank *p* = 0.018) (Figure 3).

### 3.4. Prognostic Factors in the Combined Resectable Cohort

The LR group (*n* = 57) and the potentially resectable NLR group (*n* = 27) were combined to form a cohort of 84 patients considered resectable. During follow-up, 44 deaths occurred.

In univariate Cox regression analysis, liver resection was associated with longer OS (HR 0.46, 95% CI 0.24–0.89, *p* = 0.020). Metachronous presentation was also associated with longer OS (HR 0.52, 95% CI 0.28–0.97, *p* = 0.038), whereas ≥2 liver metastases (HR 1.89, 95% CI 1.02–3.50, *p* = 0.043) and bilobar disease (HR 2.10, 95% CI 1.11–4.00, *p* = 0.023) were associated with shorter OS (Table 3).

Age, sex, primary tumor location, largest metastasis > 5 cm, extrahepatic metastases, and CEA were not significantly associated with OS.

In multivariable Cox proportional hazards analysis, liver resection remained independently associated with longer OS (adjusted HR 0.40, 95% CI 0.20–0.81, *p* = 0.011), as did metachronous presentation (adjusted HR 0.43, 95% CI 0.22–0.84, *p* = 0.013). The number of liver metastases (adjusted HR 1.34, 95% CI 0.44–4.11, *p* = 0.608) and bilobar disease (adjusted HR 1.22, 95% CI 0.39–3.83, *p* = 0.736) were not independently associated with OS (Table 4).

### 3.5. Prognostic Factors in the NLR Group

In multivariable Cox proportional hazards analysis of the NLR group, age < 65 years was independently associated with longer OS (adjusted HR 0.663, 95% CI 0.458–0.960, *p* = 0.0295). Absence of extrahepatic metastases was also independently associated with longer OS (adjusted HR 0.637, 95% CI 0.417–0.973, *p* = 0.0368) (Table 5).

### 3.6. Prognostic Factors After Liver Resection

Among the 57 patients who underwent liver resection, univariate Cox regression analysis identified colon primary (HR 0.22, 95% CI 0.07–0.67, *p* = 0.008), simultaneous colorectal and liver resection (HR 3.02, 95% CI 1.35–6.74, *p* = 0.007), synchronous CRLM (HR 2.39, 95% CI 1.04–5.47, *p* = 0.040), and positive resection margin (R1) (HR 3.40, 95% CI 1.45–8.00, *p* = 0.005) as factors significantly associated with OS (Table 6). Other evaluated factors were not significantly associated with OS.

## 4. Discussion

This study evaluated real-world treatment allocation and survival in patients with CRLM treated at a tertiary-care center in Thailand. A key finding was that 27 patients who did not undergo liver resection met predefined criteria for potential resectability on retrospective reassessment by two hepatobiliary surgeons. All 27 had undergone resection of the primary colorectal tumor, yet none had been referred for specialist hepatobiliary surgical assessment. These patients accounted for 13.0% of all NLR patients and 40.3% of NLR patients without extrahepatic metastases. This finding highlights a potential gap between technical resectability, specialist referral, and actual treatment allocation in this clinical setting.

The survival outcome after liver resection in the present study was favorable. Median OS was 59 months, and the 5-year OS rate was 49.1%. These results were comparable with previously reported surgical outcomes from Japan and other countries [3,4]. Contemporary guidelines recommend liver resection, with or without perioperative systemic therapy, for patients in whom complete treatment of liver metastases can be achieved [1,2]. However, the simple comparison between the entire LR and NLR groups should be interpreted cautiously because the NLR group had substantially more advanced disease. Five or more liver metastases, bilobar disease, and extrahepatic metastases were significantly more frequent in the NLR group. Therefore, the survival difference between the whole LR and NLR groups cannot be interpreted as a treatment effect of liver resection. The European Society for Medical Oncology guideline similarly emphasizes multidisciplinary management and integration of systemic and local treatment strategies for metastatic colorectal cancer [16]. Long-term survival after hepatectomy for CRLM has been demonstrated across multiple surgical series, including reports of durable 10-year survival in selected patients [17,18,19,20].

A more informative analysis focused on the 27 potentially resectable NLR patients. These patients did not have an extremely high liver tumor burden: none had five or more liver metastases, 88.9% had tumors ≤ 5 cm, and 59.3% had unilobar disease. When these 27 patients were compared with the 57 patients who underwent liver resection, no significant differences were observed in the measured baseline factors, including age, sex, synchronous presentation, bilobar disease, tumor size, and serum CEA level. Median OS was 29 months in the potentially resectable NLR group and 59 months in the LR group (log-rank *p* = 0.018). Nevertheless, similarity in measured variables does not eliminate treatment-selection bias or residual confounding by unmeasured clinical factors.

In the combined cohort of 84 patients considered resectable, liver resection remained associated with longer OS after adjustment for synchronous or metachronous presentation, number of liver metastases, and bilobar distribution (adjusted HR 0.40, 95% CI 0.20–0.81, *p* = 0.011). Metachronous presentation was also associated with longer OS (adjusted HR 0.43, 95% CI 0.22–0.84, *p* = 0.013). With 44 deaths and four covariates, the model contained approximately 11 events per variable. Important unmeasured factors may still influence both treatment allocation and survival, and this residual confounding should be considered when interpreting the adjusted estimates.

The concept of resectability is important when interpreting these results. In the present study, resectability was retrospectively assessed according to the indication criteria for liver resection in the JSCCR Guidelines 2024 [11], together with assessment of liver functional reserve and the permissible extent of hepatectomy using the Makuuchi criteria [12,13] and CT volumetry when the anticipated future liver remnant was insufficient or borderline [14,15]. The number, size, and bilobar distribution of liver metastases were not used as absolute contraindications to surgery. This is consistent with contemporary CRLM concepts, in which complete removal of all disease and preservation of adequate liver function are more important than simple numerical cutoffs [2,5,6]. Therefore, patients with multiple or bilobar liver metastases may still be candidates for liver-directed curative-intent treatment after specialist assessment. These principles are also consistent with an international expert consensus that emphasizes technical resectability, adequate future liver remnant, and multidisciplinary selection rather than rigid limits based solely on tumor number or distribution [21]. Systemic therapy can also change resectability over time. Perioperative FOLFOX was evaluated in patients with resectable CRLM in the EORTC 40983 trial [22], while modern conversion approaches have enabled selected patients with initially unresectable liver-limited disease to undergo secondary resection [23,24,25,26]. These data support repeated multidisciplinary reassessment during systemic treatment rather than treating resectability as a fixed baseline classification.

The multivariable analysis in the entire NLR group showed that age < 65 years and absence of extrahepatic metastases were independently associated with longer survival. These findings may help identify patients with a relatively favorable prognosis among patients who did not undergo liver resection. However, these two factors alone do not define resectability. In particular, the absence of extrahepatic metastases does not mean that all liver metastases can be completely resected. For this reason, we separately reassessed the imaging and clinical history of patients without extrahepatic metastases.

Previous reports have also shown that specialist assessment can change the treatment strategy for CRLM. Young et al. reported that, among patients considered fit but inoperable by local colorectal teams, 29% were considered operable after specialist liver review, and a further 15% had equivocal imaging [7]. Jones et al. showed that specialist reassessment of patients receiving palliative chemotherapy could identify patients who might otherwise have been denied potentially curative treatment [8]. Variation in surgical referral has also been reported, and misconceptions regarding bilobar disease, tumor number, and tumor size may influence referral decisions [9]. More recently, a Dutch expert panel reported that some patients considered resectable did not receive local treatment and that lack of referral to a liver surgeon was an important reason [10]. In our cohort, all 27 patients retrospectively classified as potentially resectable had undergone primary colorectal tumor resection by the colorectal surgical team but had not been referred for hepatobiliary surgical assessment before receiving palliative systemic therapy. Thus, the observed gap appears to have occurred at the referral stage rather than after specialist hepatobiliary evaluation. However, the specific reason why referral did not occur was not consistently documented for each patient, and our findings should not be interpreted as proof that an individual treatment decision was inappropriate. Rather, they identify a potential opportunity to strengthen referral pathways and multidisciplinary reassessment for patients with liver-limited CRLM.

The present study provides real-world information from Thailand and Southeast Asia. Thailand has developed universal health coverage and a broad referral-based health-care system with substantial investment in district, provincial, and tertiary referral hospitals [27]. The present study was performed at Bhumibol Adulyadej Hospital, a tertiary-care hospital in Bangkok. Therefore, our results should not simply be interpreted as a problem caused by treatment in a small hospital or a poorly developed healthcare system. Rather, the findings suggest that a gap between technical resectability and actual treatment allocation may occur even in a tertiary-care setting. The clinically relevant question is whether patients with liver-limited CRLM receive timely evaluation by an experienced hepatobiliary surgeon and repeated reassessment during the treatment course. Because referral pathways, specialist availability, and treatment resources vary among institutions, multicenter studies are needed to determine how frequently similar referral gaps occur in other settings. Population-based data from Sweden have likewise shown substantial heterogeneity in the incidence, metastatic pattern, management, and survival of CRLM in routine practice [28].

Among patients who underwent liver resection, several factors were associated with OS in univariate analysis, including colon primary, simultaneous colorectal and liver resection, synchronous CRLM, and positive resection margin. Positive resection margin was associated with poorer OS (HR 3.40, 95% CI 1.45–8.00, *p* = 0.005). Complete removal of macroscopic disease remains an important principle in surgery for CRLM. For selected patients who are not surgical candidates, other liver-directed treatments such as radiofrequency ablation have also been used; Facciorusso et al. reported outcomes in 127 patients with unresectable CRLM treated with percutaneous radiofrequency ablation [29]. However, the prognostic-factor analysis in the present surgical cohort included only 57 patients and was secondary; these findings should not be considered the main result of this study. In synchronous CRLM, the optimal sequence of colorectal and liver surgery remains individualized; simultaneous, staged colorectal-first, and liver-first strategies have all been used in selected patients [30].

This study has several limitations. First, its retrospective, single-center design is subject to treatment-selection bias and residual confounding; although the key Cox model was limited to four covariates for 44 deaths, unmeasured clinical and treatment-related factors remain. Second, resectability was reassessed retrospectively by two hepatobiliary surgeons using available imaging and clinical information, with the final classification reached by consensus. Because the assessment was not independent or blinded and was performed after the original treatment decisions, observer and hindsight bias cannot be excluded, and formal interobserver agreement was not assessed. Third, although all 27 potentially resectable NLR patients had undergone primary colorectal tumor resection without subsequent referral for hepatobiliary surgical assessment, the specific reasons why referral did not occur were not consistently documented; therefore, these patients should not be labeled definitively as “missed opportunities.” Fourth, available systemic-treatment information showed that these 27 patients received palliative chemotherapy with regimens including capecitabine, XELOX, FOLFOX4, and FOLFIRI, and no conversion to liver resection occurred. However, detailed treatment response, duration, sequencing, and timing were not systematically available for comparative analysis, and targeted therapy was not analyzed because its use was uncommon during the study period. Finally, survival comparisons are susceptible to immortal-time bias because patients classified in the LR group necessarily survived until surgery. These limitations should be considered when interpreting the observed survival associations.

Despite these limitations, the present study has an important clinical message. Favorable long-term survival can be achieved in selected patients who undergo liver resection, but the main real-world observation is that some patients who met contemporary resectability criteria on specialist reassessment did not undergo liver resection and, in this cohort, had not been referred for hepatobiliary surgical assessment after resection of the primary tumor. Early referral to a hepatobiliary surgeon and repeated multidisciplinary reassessment may help ensure that patients with liver-limited CRLM are evaluated for all potentially curative-intent options. Future prospective studies should systematically record the timing and reasons for surgical referral or non-referral, systemic-treatment details, changes in resectability over time, and multidisciplinary decision-making.

## 5. Conclusions

In this real-world Thai cohort, a subgroup of non-resected patients with liver-limited CRLM met predefined criteria for potential resectability on retrospective specialist reassessment. All 27 of these patients had undergone primary colorectal tumor resection but had not been referred for hepatobiliary surgical assessment. Among patients considered resectable, liver resection was associated with longer OS. These findings highlight the importance of timely hepatobiliary referral and repeated multidisciplinary reassessment to identify patients who may be candidates for curative-intent liver resection.

## Figures and Tables

**Figure 1:**
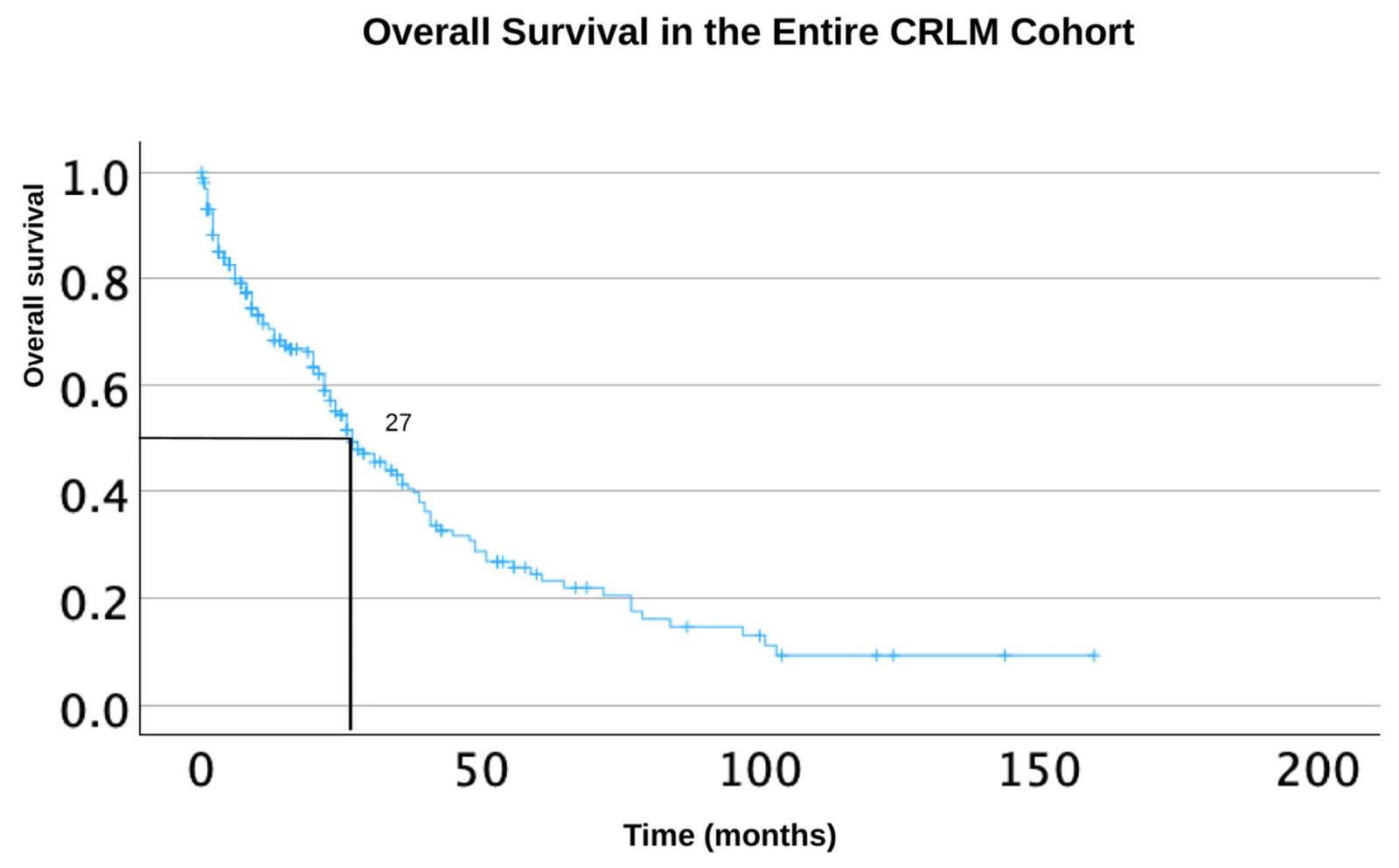
Kaplan–Meier curve for overall survival in the entire cohort (*n* = 265). Median overall survival was 27 months (95% CI, 21.0–32.9 months).

**Figure 2:**
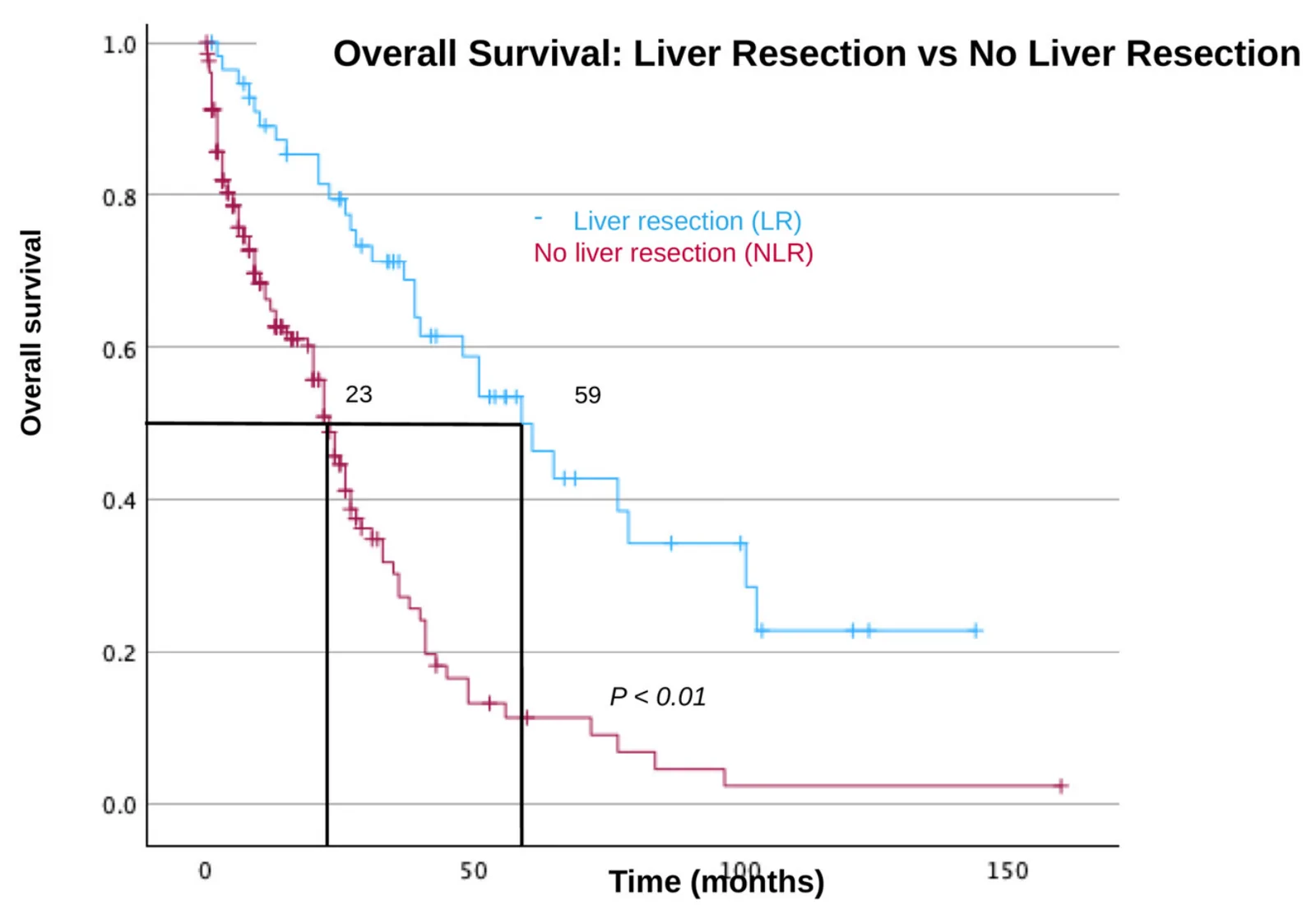
Kaplan–Meier curves for overall survival according to liver resection status. Median overall survival was 59 months (95% CI, 42.74–75.26 months) in the liver-resection group and 23 months (95% CI, 19.21–26.79 months) in the no-liver-resection group (log-rank *p* <0.01). This unadjusted comparison is descriptive.

**Figure 3:**
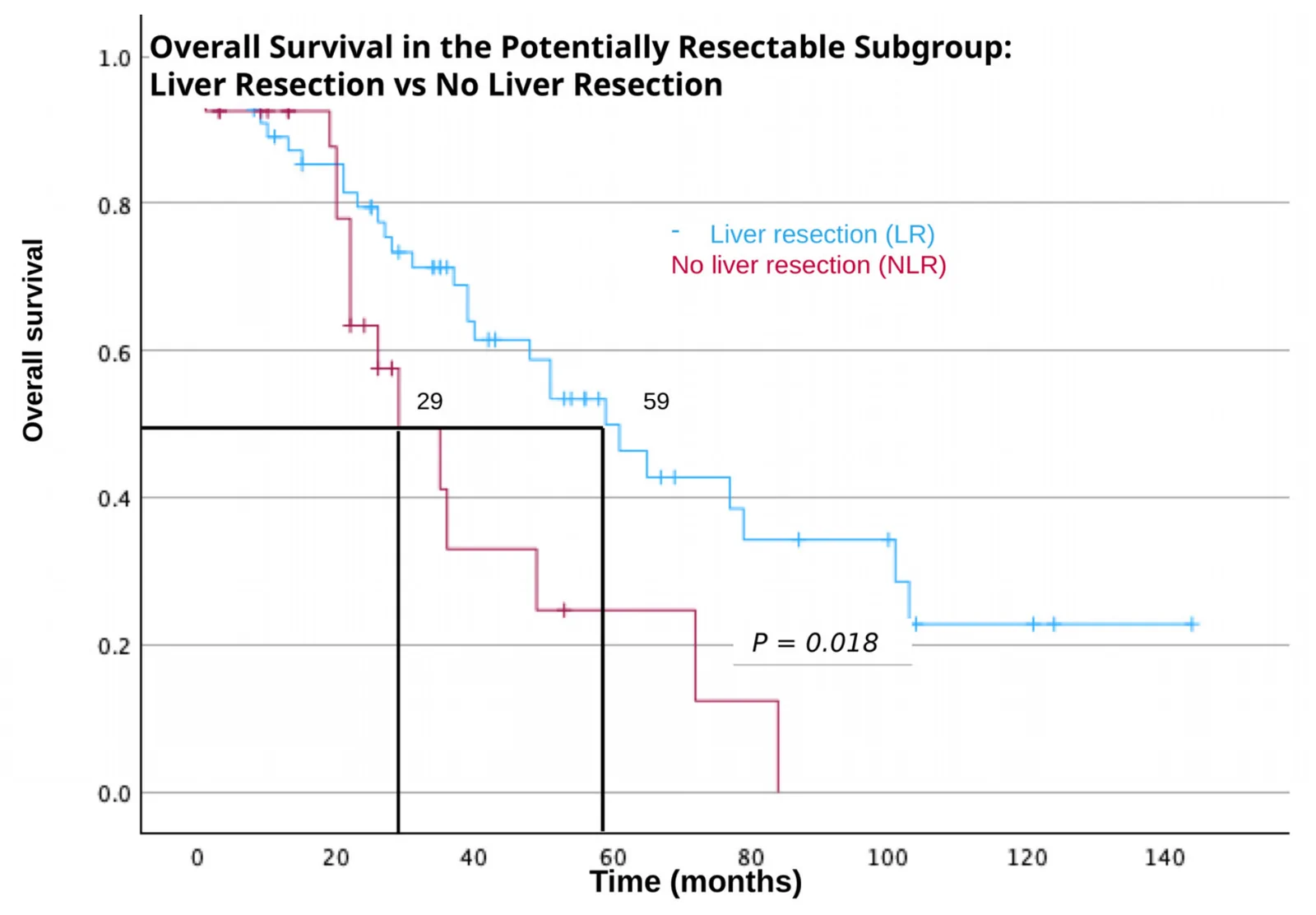
Kaplan–Meier curves for overall survival comparing patients who underwent liver resection and potentially resectable patients who did not undergo liver resection. Median overall survival was 59 months (95% CI, 42.74–75.26 months) in the liver-resection group (*n* = 57) and 29 months (95% CI, 16.02–41.98 months) in the potentially resectable no-liver-resection group (*n* = 27) (log-rank *p* = 0.018). The potentially resectable subgroup was identified by retrospective specialist reassessment according to the predefined resectability criteria.

**Table 1:** Baseline clinical characteristics and survival outcomes according to liver resection status.

		LR	NLR	*p*-Value
Male		35 (61.4)	117 (56.3)	0.49
Age (years)		64.8 ± 11.1	62.8 ± 13.2	0.177
CEA (ng/mL)		327.2 ± 1344.7	546.6 ± 2168.1	0.407
Colon		53 (93.0)	206 (99)	0.009
Left colon		32 (57)	133 (63.9)	0.282
S-CRLM		39 (68.4)	164 (78.8)	0.100
Number ≥ 5 CRLM		4 (7.0)	152 (73.1)	<0.001
Size ≤ 5 cm		44 (77.2)	117 (56.3)	0.004
Bilateral		14 (24.6)	165 (79.3)	<0.001
Extrahepatic		5 (8.8)	141 (67.8)	<0.001
Blood loss		231.1 ± 88.3	-	
Operative time		238.7 ± 89.5	-	
Survival time (months)		45.0 ± 33.7	15.6 ± 18.8	<0.001
Survival (year)				
	1	51 (89.5)	134 (64.4)	<0.001
	3	41 (71.9)	57 (27.4)	<0.001
	5	28 (49.1)	24 (11.5)	<0.001

Data are presented as *n* (%) or mean ± standard deviation (SD), as appropriate. LR, liver resection; NLR, no liver resection; CRLM, colorectal liver metastases; S-CRLM, synchronous colorectal liver metastases; CEA, carcinoembryonic antigen; SD, standard deviation.

**Table 2:** Baseline characteristics of patients who underwent liver resection and potentially resectable patients who did not undergo liver resection.

Characteristic	Retrospectively Resectable, Not Resected (*n* = 27)	Liver Resection (*n* = 57)	*p*-Value
Male, *n* (%)	15 (55.6)	35 (61.4)	0.641
Age, mean ± SD	67.6 ± 13.4	64.8 ± 11.1	0.312
Synchronous presentation, *n* (%)	13 (48.1)	39 (68.4)	0.094
Bilobar disease, *n* (%)	11 (40.7)	14 (24.6)	0.201
Tumor > 5 cm, *n* (%)	3 (11.1)	13 (22.8)	0.247
CEA (ng/mL), median (IQR)	10.0 (4.5–30.0)	10.5 (4.6–49.0)	0.958
Death during follow-up, *n* (%)	15 (55.6)	29 (50.9)	0.816

**Table 3:** Univariate Cox regression analysis of factors associated with overall survival among patients with resectable colorectal liver metastases (liver resection, *n* = 57; retrospectively resectable but non-resected, *n* = 27; combined *n* = 84, 44 deaths).

Predictor (Reference)	HR	95% CI	*p*
Liver resection (vs. not resected)	0.46	0.24–0.89	0.020
Age < 65 years (vs. ≥65)	0.75	0.41–1.38	0.355
Male sex	1.04	0.57–1.91	0.902
Left-sided primary (vs. right)	0.99	0.54–1.83	0.986
Metachronous presentation (vs. synchronous)	0.52	0.28–0.97	0.038
≥2 liver lesions (vs. 1)	1.89	1.02–3.50	0.043
Largest metastasis > 5 cm	1.02	0.39–2.66	0.965
Bilobar disease (vs. unilobar)	2.10	1.11–4.00	0.023
Extrahepatic metastases	1.75	0.62–4.94	0.288
CEA (log-transformed, continuous)	0.98	0.81–1.17	0.808

HR: hazard ratio; CI: confidence interval; CEA: carcinoembryonic antigen.

**Table 4:** Multivariable Cox regression analysis of overall survival, liver-resection group (*n* = 57) vs. retrospectively resectable non-resected group (*n* = 27), combined *n* = 84.

Predictor (Reference)	Adjusted HR	95% CI	*p*
Liver resection (vs. not resected)	0.40	0.20–0.81	0.011
Metachronous presentation (vs. synchronous)	0.43	0.22–0.84	0.013
≥2 liver lesions (vs. 1)	1.34	0.44–4.11	0.608
Bilobar disease (vs. unilobar)	1.22	0.39–3.83	0.736

HR: hazard ratio; CI: confidence interval.

**Table 5:** Multivariable Cox proportional hazards analysis among patients who did not undergo liver resection.

Predictor	Adjusted HR	95% CI	*p*-Value
Age < 65 years	0.663	0.458–0.960	0.0295
Absence of extrahepatic metastases	0.637	0.417–0.973	0.0368

HR: hazard ratio; CI: confidence interval.

**Table 6:** Univariate Cox regression analysis in the CRLM liver-resection group (*n* = 57).

Predictor (Reference)	HR	95% CI	*p*
Male sex	0.84	0.40–1.77	0.65
Primary tumor T1–2	2.08	0.49–8.88	0.32
Regional node N1–2	1.70	0.71–4.08	0.23
Colon primary	0.22	0.07–0.67	0.008
Simultaneous colorectal + liver resection	3.02	1.35–6.74	0.007
Synchronous CRLM	2.39	1.04–5.47	0.04
≥2 liver metastases	1.83	0.84–3.95	0.13
Unilobar disease	0.48	0.21–1.10	0.08
Largest metastasis > 5 cm	1.46	0.52–4.07	0.47
Extrahepatic metastases	2.17	0.75–6.32	0.16
Neoadjuvant chemotherapy	1.53	0.52–4.47	0.44
Positive resection margin, R1	3.40	1.45–8.00	0.005

HR: hazard ratio; T1–2: primary tumor staging 1 and 2; N1–2: lymph node staging 1 and 2.

## Data Availability

The datasets used and analyzed during the current study are not publicly available due to patient privacy and institutional regulations but are available from the corresponding author upon reasonable request.
